# Comparison of the epidemiology of laboratory-confirmed influenza A and influenza B cases in Manitoba, Canada

**DOI:** 10.1186/s12889-015-1351-z

**Published:** 2015-01-30

**Authors:** Aynslie M Hinds, Songul Bozat-Emre, Paul Van Caeseele, Salaheddin M Mahmud

**Affiliations:** Vaccine and Drug Evaluation Centre, Department of Community Health Sciences, Faculty of Health Sciences, University of Manitoba, S111 - 750 Bannatyne Avenue, Winnipeg, Manitoba R3E 0W3 Canada; Department of Medical Microbiology, Faculty of Medicine, University of Manitoba, Winnipeg, Manitoba Canada; Cadham Provincial Laboratory, Winnipeg, Manitoba Canada; Faculty of Pharmacy, University of Manitoba, Winnipeg, Manitoba Canada

**Keywords:** Epidemiology, Influenza A, Influenza B, Vaccine

## Abstract

**Background:**

Despite the public health significance of annual influenza outbreaks, the literature comparing the epidemiology of influenza A and B infections is limited and dated and may not reflect recent trends. In Canada, the relative contribution of influenza A and B to the burden of morbidity is not well understood. We examined rates of laboratory-confirmed cases of influenza A and B (LCI-A and LCI-B) in the Canadian province of Manitoba between 1993 and 2008 and compared cases of the two types in terms of socio-demographic and clinical characteristics.

**Methods:**

Laboratory-confirmed cases of influenza A and B in Manitoba between 1993 and 2008 were identified from the Cadham Provincial Laboratory (CPL) Database and linked to de-identified provincial administrative health records. Crude and age-adjusted incidence rates of LCI-A and LCI-B were calculated. Demographic characteristics, health status, health service use, and vaccination history were compared by influenza type.

**Results:**

Over the study period, 1,404 of LCI-A and 445 cases of LCI-B were diagnosed, corresponding to an annual age-standardized rate of 7.2 (95% CI: 6.5-7.9) for LCI-A and 2.2 (CI: 1.5 – 3.0) per 100,000 person-years for LCI-B. Annual rates fluctuated widely but there was less variation in the LCI-B rates. For LCI-A, but not LCI-B, incidence was inversely related to household income. Older age, urban residence and past hospitalization were associated with increased detection of LCI-A whereas receipt of the influenza vaccine was associated with decreased LCI-A detection. Once socio-demographic variables were controlled, having a pre-existing chronic disease or immune suppression was not related to influenza type.

**Conclusion:**

Influenza A and B affected different segments of the population. Older age was associated with increased LCI-A detection, but not with pre-existing chronic diseases. This information may be useful to public health professionals in planning and evaluating new and existing seasonal influenza vaccines.

## Background

Annual epidemics of influenza are an important public health problem globally and in Canada [1]. Each year, 10-25% of the Canadian population become infected with influenza [2]. Most of these infections are typically asymptomatic or associated with a mild self-limiting illness [3]. However, influenza can cause severe illness leading to hospitalization and death, especially among the very young, the elderly and those with underlying chronic conditions [3,4]. It has been estimated that on average about 4,000 influenza-related deaths occur in Canada each year [5].

Influenza is caused by small negative-sense single stranded RNA viruses that belong to one of three genera (Influenza virus A, B and C) of the family Orthomyxoviridae [6]. Influenza C, which is typically associated with mild illness in children [3], is of little clinical or epidemiologic significance. Although influenza A and B types are indistinguishable morphologically and share similar clinical presentations and epidemiological features, they differ in several important ways. Unlike influenza A, which infects many bird and mammalian species, the influenza B virus only infects humans (and occasionally seals [7]). Because of the lack of animal reservoir, influenza B has a lower rate of antigenic drift and does not cause pandemics, although it could still cause significant epidemics [8].

Although it is widely accepted that influenza B viruses are more likely to infect children and to cause milder illness than are influenza A viruses [9], the literature comparing the epidemiology of influenza A and B infections is limited and dated and may not reflect recent trends [10]. In Canada, the relative contribution of influenza A and B to the burden of morbidity is not well understood.

The objectives of this population-based study were to examine rates of laboratory-confirmed cases of influenza A and B in the Canadian province of Manitoba between 1993 and 2008 and to compare cases of these two influenza types in terms of socio-demographic and clinical characteristics.

## Methods

### Data sources

This study was conducted using de-identified records obtained by linking the electronic database of Cadham Provincial Laboratory (CPL) with other Manitoba Health (MH) administrative databases, housed at the Manitoba Centre for Health Policy. The study was approved by the Research Ethics Board of the University of Manitoba and the Health Information Privacy Committee of MH.

MH is the publicly funded government department providing comprehensive health insurance, including coverage for laboratory, hospital and outpatient physician services, to the province’s 1.2 million residents. Coverage is universal (there is no eligibility distinction based on age or income) and participation rates are very high (>99%) [11]. Only RCMP and military personnel, whose health benefits are fully covered by the federal government, are not included [12]. For administrative purposes, MH maintains several centralized electronic databases that can be linked using a unique health services number (PHIN). The completeness and accuracy of the MH databases are well established [13,14], and these databases have been used extensively, by our team and others, in studies of influenza surveillance and vaccine evaluation [15-17].

### Identification of influenza A and B cases

We used the database of CPL, the province’s only virology laboratory, to identify all individuals diagnosed in Manitoba with laboratory-confirmed influenza (LCI) A or B between 1993 and 2008 (the study period). This database has stored results of all influenza testing performed in the province since the early 1980s [18]. Over the study period, influenza testing was mostly performed using viral cultures or more recently Poymerase Chain Reaction (PCR) and very occasionally using other tests including antigen detection, fluorescence microscopy and egg inoculation.

For the purpose of this analysis, multiple positive tests for the same person were counted as a single incident infection if they were performed within 13 days of each other. Positive tests performed 14 or more days apart were considered as separate cases. Since the date of symptom onset was not available in the laboratory data, the specimen collection date was used as the index date (*epidate*) in this study. In instances where the specimen date was missing, the date the specimen was received or tested was used instead.

To be included in the analysis, individuals with LCI had to be residents of Manitoba and have MH coverage when they were tested. Eligibility was ascertained by linking the CPL database, using the scrambled PHIN, with MH Population Registry, a continuously updated registry that stores basic demographic information on all insured Manitobans, and gathers information on dates and reasons for the initiation and termination of health care coverage (e.g., birth, migration in or out of province and death).

### Measurement of covariates

Sex, age, and region of residence at the index date were obtained from the MH Population Registry. Household income quintiles, measured at the level of the census dissemination area, were determined using the 2006 Canadian census data. Information on propensity to seek health care and on relevant pre-existing health conditions was obtained from hospital and physician claims databases. Since 1971, the Hospital Abstracts database recorded virtually all services provided by hospitals in the province, including admissions and day surgeries.[12] The data collected comprise demographic as well as diagnosis and treatment information including primary diagnosis and service or procedure codes, coded using the International Classification of Diseases, Ninth Revision, Clinical Modification (ICD-9-CM) before April, 2004, and the ICD-10-CA [19] (Canadian adaptation of the ICD-10 [20]) and the Canadian Classification of Health Interventions (CCI) [21] afterwards. The Medical Services database, also in operation since 1971, collects similar information, based on physician fee-for-service or shadow billing, on services provided by physicians in offices, hospitals and outpatient departments across the province [12]. Each billing record includes a tariff code and a 3-digit ICD-9 code which identifies the principal diagnosis or main reason for the visit.

We used previously validated algorithms, based on the frequency of certain International Classification of Diseases’ codes, to identify chronic diseases such as asthma, diabetes etc. (See Appendix 1 for the definitions of these conditions) [22,23]. Immunosuppression was defined as having a diagnosis of HIV/AIDS, other immune deficiency disorders or cancer (other than non-melanoma skin cancer), or receiving prescriptions for immunosuppressive drugs [24]. Information on the use of immune suppressants was obtained from DPIN, the comprehensive database of all out-of-hospital prescriptions dispensed in Manitoba since 1995. In addition, Charlson comorbidity scores were calculated using an algorithm validated for electronic databases [25].

Information on the receipt of all vaccines, including the seasonal influenza vaccines (Trivalent Influenza Vaccines (TIV)) and pneumococcal vaccines was obtained from the Manitoba Immunization Monitoring System (MIMS), the population-based province-wide registry recording all immunizations administered to Manitoba residents since 1988 [26]. Information, including vaccine type and date of immunization, is captured for each immunization event either through direct data entry for vaccines administered by public health staff or using physician claims data for vaccines administered by physicians [27].

### Statistical analysis

Annual age-standardized incidence rates were calculated separately for influenza A and B cases using population estimates obtained from the MH Population Registry as the denominator and the 2006 Canadian population as the standard population. Age-standardized rates were also calculated for subsets of the population defined by gender, area of residence, and income quintiles.

We described and compared the demographic and clinical profiles of the influenza A and B cases, and used unconditional logistic regression models to estimate the odds ratio (OR), and 95% confidence intervals (95%CI), for the association between the detection of LCI-A and B infections and several socio-demographic and clinical factors. Selection of variables for inclusion in the model was guided by what is known from the literature and by the extent that the inclusion of the variable improved model fitness assessed using a likelihood ratio test.

## Results

Over the study period, a total of 1849 LCI cases met the eligibility criteria: 1404 (75.9%) were influenza A whereas the other 445 were influenza B corresponding to an age-standardized rate of 7.2 cases per 100,000 person-years (95% CI 6.5-7.9) for LCI-A and 2.2 per 100,000 (95% CI 1.5-3.0) for LCI-B (Table 1). LCI-A age-specific rates were generally higher than the LCI-B rates especially among infants and those 75 or older. For both LCI-A and B, the age-specific rate was highest in infants, and tended to decrease with age and then increase again among those 75 or older.

**Table 1:** **Crude and age-standardized rates (per 100,000 person-years) of laboratory-confirmed influenza A and B cases by certain socio-demographic characteristics (1993–2008)**

	**Total person-years**	**Influenza A**					**Influenza B**				
		**Number of events**	**Crude**		**Age-standardized**		**Number of events**	**Crude**		**Age-standardized**	
			**Rate**	**95% CI**	**Rate**	**95% CI**		**Rate**	**95% CI**	**Rate**	**95% CI**
*Overall*	18,559,978	1,404	7.6	7.2 - 8.0	7.2	6.5 - 7.9	445	2.4	2.2 - 2.6	2.2	1.5 - 3.0
**Age group (years)**											
<=1	478,951	323	67.4	60.5-75.2			94	19.6	16.0-24.0		
2 to 4	741,193	68	9.2	7.2-11.6			32	4.3	3.1-6.1		
5 to 9	1,292,298	58	4.5	3.5-5.8			46	3.6	2.7-4.8		
10 to 14	1,333,125	62	4.7	3.6-6.0			64	4.8	3.8-6.1		
15 to 24	2,581,745	68	2.6	2.1-3.3			41	1.6	1.2-2.2		
25 to 44	5,379,985	142	2.6	2.2-3.1			57	1.1	0.8-1.4		
45 to 64	4,227,965	115	2.7	2.3-3.3			31	0.7	0.5-1.0		
65 to 74	1,290,068	66	5.1	4.0-6.5			7	0.5	0.3-1.1		
75+	1,234,648	502	40.7	37.3-44.4			73	5.9	4.7-7.4		
**Sex**											
Female	9,412,486	758	8.1	7.5 - 8.6	7.4	6.3 - 8.4	245	2.6	2.3 - 3.0	2.4	1.4 - 3.5
Male	9,147,492	646	7.1	6.5 - 7.6	6.8	5.8 - 7.9	200	2.2	1.9 - 2.5	1.9	0.9 - 3.0
**Locality of residence**											
Rural	7,268,040	604	8.3	7.7 - 9.0	7.7	6.5 - 8.9	240	3.3	2.9 - 3.7	2.9	1.7 - 4.1
Urban	11,291,938	800	7.1	6.6 - 7.6	6.9	5.9 - 7.8	205	1.8	1.6 - 2.1	1.7	0.8 - 2.7
**Area of residence**											
North	1,138,287	126	11.1	9.3 - 13.2	8.4	5.1 - 11.6	42	3.7	2.7 - 5.0	2.3	0.3 - 7.1
South	17,421,691	1,278	7.3	6.9 - 7.7	7.0	6.2 - 7.8	403	2.3	2.1 - 2.6	2.2	1.4 - 2.9
**Income quintiles**											
Q1 (lowest)	3,704,516	385	10.4	9.4 - 11.5	8.7	7.0 - 10.3	108	2.9	2.4 - 3.5	2.3	0.6 - 4.0
Q2	3,838,620	249	6.5	5.7 - 7.3	5.9	4.3 - 7.5	74	1.9	1.5 - 2.4	1.8	0.7 - 4.7
Q3	3,722,216	225	6.0	5.3 - 6.9	5.7	4.0 - 7.3	108	2.9	2.4 - 3.5	2.8	1.1 - 4.4
Q4	3,707,649	160	4.3	3.7 - 5.0	4.3	2.7 - 6.0	60	1.6	1.3 - 2.1	1.5	0.6 - 5.0
Q5 (highest)	3,315,922	135	4.1	3.4 - 4.8	4.4	2.6 - 6.1	48	1.4	1.1 - 1.9	1.3	0.4 - 5.7
Unknown	271,055	250	92.2	81.5 - 104.4	59.7	53.1 - 66.4	47	17.3	13.0 - 23.1	13.9	0.4 - 6.1

**Figure 1:**
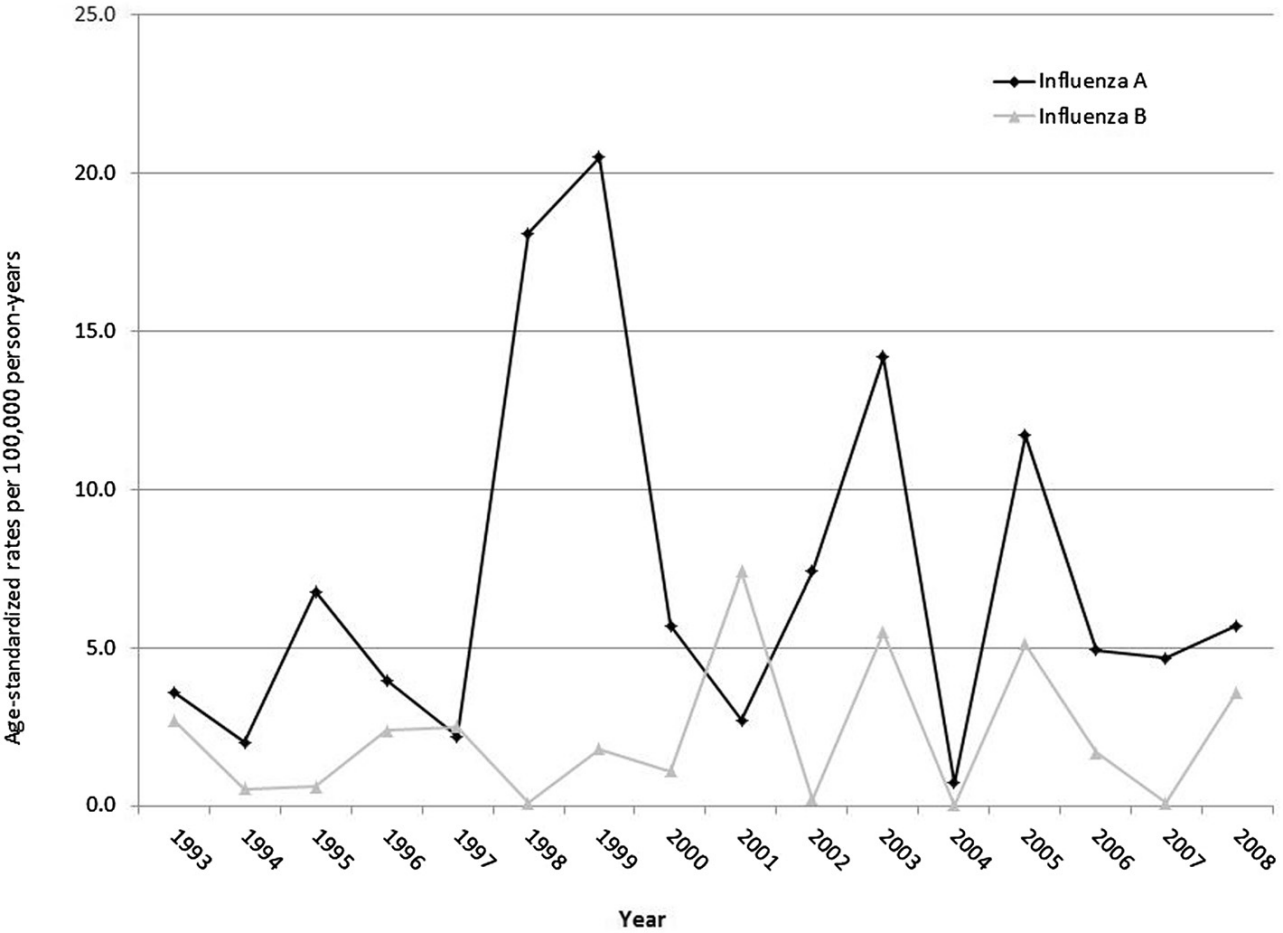
**Annual age-standardized rates by laboratory-confirmed influenza type, Manitoba 1993–2008.**

Annual rates fluctuated widely but there was less fluctuation in the annual rates for LCI-B compared to the typically much higher LCI-A rates (Figure 1). The annual age-standardized rates of LCI-A ranged from 0.7 in 2004 to 21.0 per 100,000 person-years in 1999. Meanwhile, the annual age-standardized rates for LCI-B ranged from <0.5 in 1998, 2002, and 2007 to 7.8 per 100,000 person-years in 2001.

LCI-B cases were younger with a median (range) age of 13 (3–43) years compared to 42 (2–83) years for LCI-A. For both LCI-A and B, the age-standardized rates did not vary much by gender or area of residence. For LCI-A, but not LCI-B, there was an income gradient, with age-standardized rates decreasing from 8.7 (7.0 - 10.3) among those residing in the poorest areas to 4.4 (2.6 - 6.1) per 100,000 person-years among those residing the wealthiest areas. For both LCI-A and B, the highest rates were in the unknown income quintile category, which includes individuals residing in personal care homes and similar long-term care facilities.

The clinical characteristics of the laboratory-confirmed influenza A and B cases are presented in Table 2. Compared to LCI-B cases, a larger proportion of LCI-A cases had pre-existing chronic diseases including asthma, cardiovascular diseases, chronic obstructive pulmonary disease and diabetes. They were also more likely to have been hospitalized or seen a physician during the previous 5 years. While there was no significant difference between the two groups in receiving the TIV, a larger proportion of LCI-A cases were eligible to receive the TIV but did not receive it. Lastly, 36.2% of LCI-A cases received a pneumococcal vaccine compared to 25.2% of LCI-B cases.

**Table 2:** **Clinical characteristics of laboratory-confirmed influenza A and B cases (1993–2008)**

	**Influenza A (n = 1,404)**		**Influenza B (n = 445)**		
	**N**	**%**	**N**	**%**	**P-value** ^**†**^
**Asthma**	141	10.0	51	11.5	0.393
**Any cardiovascular disease**	585	41.7	110	24.7	<.001
**Chronic obstructive pulmonary disease**	242	17.2	45	10.1	<.001
**Diabetes**	133	9.5	20	4.5	<.001
**Immunosuppressed**	252	17.9	61	13.7	0.038
Any chronic disease*	625	44.5	145	32.6	<.001
**Mean Charlson index (SD)**	1	1.3	0	0.9	<.001
**Any hospital admissions in last 5 years**	1,046	74.5	252	56.6	<.001
Median hospital admission in last 5 years (IQR)	2	0 - 3	1	0 - 2	<.001
**Had ≥37 physician visits in last 5 years**	703	50.1	154	34.6	<.001
Median physician visit in last 5 years (IQR)	37	0 - 73	0	0 - 47	<.001
**Received the seasonal influenza vaccine**	242	17.2	64	14.4	0.158
Timing of receipt of the seasonal influenza vaccine					0.100
1-13 days before index date	10	0.7	0	0.0	
≥14 days before the index date	232	16.5	64	14.4	
**Recommended receipt of the seasonal influenza vaccine**					<.001
Not recommended	305	21.7	187	42.0	
Recommended and received	218	15.5	54	12.1	
Recommended but not received	881	62.7	204	45.8	
**Received a pneumococcal vaccine**	508	36.2	112	25.2	<.001

^†^P-values from a Chi-squared test.

*Defined as diagnosis with one of the following diseases: diabetes, chronic obstructive pulmonary disease, asthma, ischemic heart disease, chronic renal failure, or cancer (excluding non-melanoma skin cancer).

Table 3 presents the results from unadjusted models of several demographic and clinical characteristics as well as adjusted models that included terms for age group, gender, region of residence and income quintiles. Compared to infants, children between the ages of 2 and 9 years were less likely to test positive for influenza A than influenza B (adjusted OR = 0.5 [95% CI 0.3-0.9] for the 2- to 4-years-olds and OR = 0.4 [95% CI 0.3-0.7] for the 5- to 9-years-olds). On the other hand, adults aged 65 or older were more likely to test positive for influenza A, with an OR of 3.1 (95% CI 1.3-7.3) for 65- to 74-years-olds and 2.4 (95% CI 1.5-3.7) for those 75 or older. Residents in urban areas were 30% more likely to test positive for influenza A than to influenza B. Individuals who were hospitalized at least once in the last five years were significantly more likely to test positive for influenza A than B (OR = 1.7, 95% CI 1.3-2.3). Individuals that received the seasonal influenza vaccine were significantly less likely to test positive for influenza A than B (OR = 0.6, 95% CI 0.4-0.9). Once socio-demographic variables were controlled, having a pre-existing chronic disease, immune suppression or any of several specific chronic diseases were not related to influenza type (Table 3).

**Table 3:** **Effect of demographic and clinical characteristics on laboratory-confirmed influenza type**

**Variables**	**Crude**		Adjusted*	
	**OR**	**95% CI**	**OR**	**95% CI**
**Age group (years)**				
<=1	Reference group			
2 to 4	0.6	0.4 - 1.0	0.6	0.4 - 1.0
5 to 9	0.4	0.2 - 0.6	0.5	0.3 - 0.9
10 to 14	0.3	0.2 - 0.4	0.4	0.3 - 0.7
15 to 24	0.5	0.3 - 0.8	0.8	0.5 - 1.3
25 to 44	0.7	0.5 - 1.1	1.0	0.6 - 1.6
45 to 64	1.1	0.7 - 1.7	1.5	0.9 - 2.5
65 to 74	2.7	1.2 - 6.2	3.1	1.3 - 7.3
75+	2.0	1.4 - 2.8	2.4	1.5 - 3.7
**Female**	1.0	0.8 - 1.2	0.8	0.7 - 1.0
**Resides in an urban area**	1.6	1.3 - 1.9	1.3	1.1 - 1.7
**Income quintiles**				
Q1 (lowest)	Reference group			
Q2	0.9	0.7 - 1.3	1.0	0.7 - 1.5
Q3	0.6	0.4 - 0.8	0.6	0.5 - 0.9
Q4	0.7	0.5 - 1.1	0.9	0.6 - 1.4
Q5 (highest)	0.8	0.5 - 1.2	1.0	0.6 - 1.5
Unknown	1.5	1.0 - 2.2	1.0	0.6 - 1.5
**Asthma**	0.9	0.6 - 1.2	0.9	0.6 - 1.3
**Any cardiovascular disease**	2.2	1.7 - 2.8	0.8	0.5 - 1.1
**Chronic obstructive pulmonary disease**	1.9	1.3 - 2.6	1.3	0.9 - 1.9
**Diabetes**	2.2	1.4 - 3.6	1.2	0.7 - 2.0
**Immunosuppressed**	1.4	1.0 - 1.9	0.8	0.6 - 1.2
**Any chronic disease**	1.7	1.3 - 2.1	1.0	0.7 - 1.3
**Charlson index**	1.3	1.1 - 1.4	1.1	1.0 - 1.2
**Any hospital admissions in last 5 years**	2.2	1.8 - 2.8	1.7	1.3 - 2.3
**Had** ≥**37 physician visits in last 5 years**	1.9	1.5 - 2.4	1.0	0.8 - 1.4
**Received the seasonal influenza vaccine**	1.2	0.9 - 1.7	0.6	0.4 - 0.9
**Received a pneumococcal vaccine**	1.7	1.3 - 2.1	0.9	0.7 - 1.2

*Final adjusted models included each variable in conjunction with the following covariates: age, sex, locality of residence and income (see text).

## Discussion

Interest in understanding the comparative epidemiology and disease burden associated with influenza A and B infections has been growing, partially because of concerns about the effectiveness of existing TIVs in preventing influenza B infections as evidenced by recent publications from several jurisdictions showing suboptimal effectiveness against influenza B infections in some seasons [28-30]. In addition, the licensing and introduction of several quadrivalent vaccines (QIVs), targeting both influenza B lineages (Victoria and Yamagata), has sparked debate about the incremental utility and cost-effectiveness of using these vaccines instead of conventional TIVs which only targets one or the other lineage [31].

We used pre-collected population-based laboratory and administrative data to examine recent trends in the incidence of LCI-A to compare LCI-A and B cases in terms of socio-demographic and clinical profiles. Studying differences between influenza A and B infections at the population level poses significant challenges which may explain the relatively scant literature in this area. Most influenza cases are not clinically detected either because the infected persons are asymptomatic or because they decide not to seek care. Also, most cases can be managed conservatively, so a definitive laboratory diagnosis is usually not needed. Yet, understanding differences between influenza types requires a definitive laboratory diagnosis.

Confirmed cases likely represent a small minority of all those who were infected [32,33]. Therefore, our study likely underestimates the incidence of disease in the population. Also, the number of detected cases largely reflects the proportion of symptomatic patients who presented for medical care and were tested for the infection, and is, therefore, likely to be influenced by regional differences in access to medical care, physicians’ practices, laboratory testing guidelines and other factors. The extent to which these factors may bias comparisons of the demographic and clinical characteristics of laboratory-confirmed cases between the two types is not clear. Our results likely reflect the reality of clinical cases in Manitoba accurately, but may not be generalizable to other jurisdictions with drastically different healthcare systems and clinical guidelines.

We found that overall influenza A was more commonly detected. This is consistent with results from national surveillance systems [34,35] and observational studies from both temperate and tropical countries. In the Netherlands, between 1992/93 and 2006/07, influenza A was more prevalent than influenza B among sentinel patients with influenza-like illness, although there were a few seasons where influenza B was the dominant type [36]. In a meta-analysis of studies conducted in Latin America and the Caribbean, the pooled percentage of total respiratory specimens positive for influenza ranged from 4.7% and 15.4% per year between 1999 and 2008 [37]. In general, influenza A positive samples were more common than influenza B positive samples; although there were a few years in a few countries where there was a higher percentage of influenza B positive samples than influenza A positive samples [37].

In our study, after controlling various covariates, only age, urban residence, previous hospitalization, and receipt of the TIV were significantly associated with influenza type. Children 2 to 9 were less likely to test positive for influenza A compared to infants and adults 65 years and older. This could partially be a reflection of increased severity of illnesses in these age groups and therefore increased chance of detection. However, our results are consistent with widely held belief that influenza B is more likely to affect older children and young adults [9], which may be explained by observations from serologic studies that unvaccinated children accumulated natural immunity to influenza B more slowly than to influenza A [31]. In addition, there is evidence that among children, the TIV might be less effective in inducing protection against opposite-lineage influenza B strains, resulting in overall lower effectiveness against influenza B in that age group [38]. We also found that individuals who received the inactivated TIV (exclusively used in Manitoba during the study period) were significantly less likely to test positive for influenza A than B, which again is consistent with the above explanation and with the literature [39].

We saw evidence of an income gradient for influenza A, such that age-adjusted rates were progressively lower with higher average household income. This may reflect increased susceptibility to infection, higher chance of developing complications (leading to higher chance of detection) or generally increased propensity to seek healthcare among lower-income individuals. Although the publically funded Canadian healthcare systems improves access to healthcare services among lower-income individuals, there is no reason to believe that they will be more successful in negotiating obtaining medically unnecessary influenza tests than socially advantaged individuals.

Increased susceptibility is plausible. In fact, a very similar income gradient was observed in Manitoba for other infectious diseases, including respiratory infections such as tuberculosis and invasive pneumococcal disease, where laboratory testing is less discretionary [40]. Also, during the first wave of the 2009 pandemic, cumulative incidence of H1N1 infection, as measured in seroprevalence studies, was much higher in marginalized and socio-economically disadvantaged populations [41,42]. In addition, lower-income individuals in Manitoba, with higher levels of predisposing conditions, were more likely to develop severe illness requiring hospitalization [43,44], which is likely the case with seasonal influenza too. There is a dearth of studies explicitly reporting on this association. Measures of socioeconomic status were associated with the incidence of influenza-like illness in one study, although, like in our study, limited statistical power did not permit drawing firm conclusions [45]. It is worth mentioning that institutionalized people, who had the highest rates of LCI-A, were not included in the income gradient analysis.

The strengths of our study include its population-based design and relatively large sample size. Because of the availability of accurate automated databases [46], this study was less susceptible to measurement errors, for instance in determining vaccination status. The completeness and accuracy of the MH database are well established [13]. However, it is possible that some variables were not accurately measured. Misclassification of influenza type is less likely because of the use of generally accurate diagnostic tests [47]. But, we did not have information on viral subtypes because subtyping was not performed routinely on all isolates. We also did not have information on lifestyle and environmental factors, so it was not possible to assess the impact of these factors.

## Conclusions

We found that in Manitoba influenza A was more commonly detected than influenza B during the study period. Older age, urban residence and past hospitalization were associated with increased detection of LCI-A whereas receipt of the influenza vaccine was associated with decreased LCI-A detection. Pre-existing health conditions were not significantly associated with influenza type. This information may be useful to public health professionals in planning and evaluating new and existing seasonal influenza vaccines. There was also evidence of an income gradient for influenza A, which requires further exploration in future research.

## Abbreviations

CPL: Cadham Provincial Laboratory
CI: Confidence interval
MH: Manitoba Health
LCI: Laboratory-confirmed influenza
MIMS: Manitoba Immunization Monitoring System
RT-PCR: Reverse transcriptase-PCR
TIV: Trivalent influenza vaccines
